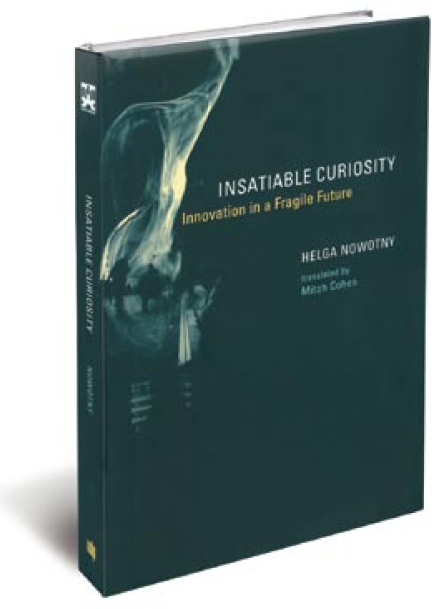# Insatiable Curiosity: Innovation in a Fragile Future

**Published:** 2009-01

**Authors:** Cyril Aydon

**Affiliations:** *Cyril Aydon is a full-time writer specializing in the history of science. His books, which include* A Book of Scientific Curiosities, A Brief Guide to Charles Darwin’s Life and Times, *and* A Brief History of Mankind*, have been published in 11 countries.*

Helga Nowotny is Vice President of the European Research Council, and Chair of the Scientific Advisory Board of Austria’s Vienna University. This slim text is a meditation on the interrelationship of scientific research and technological innovation, on the threats that societal influences pose to their successful continuation, and on the problem of reconciling the innovator’s need for freedom to follow where inquiry leads with society’s interest in defining the acceptable limits of such enquiry.

Nowotny’s central concern is with the dramatic change in the pace of scientific advance in recent decades and in the increasing tensions that result from what she terms the “privatization” of scientific research and the “democratization” of public attitudes to it. By “privatization” Nowotny means the increasing proportion of research activity accounted for by commercial and governmental (especially military) programs. By “democratization” she means the increasing involvement of the community at large, via the legislature and pressure groups, in decisions concerning the conduct and the acceptability of scientific experiment (e.g., in stem cell research), and new technologies such as genetic engineering.

Alongside this accelerating pace of innovation, there has been a striking change in the way we view the future. Nowotny contrasts the assumptions that underlay *The Limits of Growth,* the Club of Rome report that sold 30 million copies worldwide in the 1970s, with the very different attitude with which we approach such questions today. With an insouciance rooted in the apparent robustness of the new science of computer modeling, that report talked confidently about “The Future.” Just a generation later, with an awareness based on the still newer sciences of complexity and chaos, we no longer talk about “The Future”: We have had to learn the language of possible Futures.

Although Nowotny is concerned with the problems of the present and the immediate future, her argument gains perspective from her reflections on the positive characteristics of past societies that have proved successful nurseries of scientific discovery, and the negative qualities of others that were less productive of innovation.

Nowotny’s conclusions are unequivocal. There has been a permanent change in the social context of scientific enquiry and technological innovation. The tensions arising from the conflict between the drive for technological novelty and the need to guard against its possibly adverse consequences are more likely to increase than to abate, as innovation continues its headlong course. But that is no reason to erect barriers against the pursuit of the new. We need to preserve freedom of inquiry, with all its potential benefits, while increasing the vigilance with which we seek to anticipate its possibly deleterious side effects. We cannot stop the merry-go-round that began with the Scientific Revolution of the 17th century and the Industrial Revolution of the 18th. An accelerating rate of innovation is the defining characteristic of the society we have created, and we must find the nerve to live with it and devise methods of social control that will enable us to do so safely.

But in devising these controls, we need to protect researchers and designers from the narrowing influence of commercial sponsorship, with its preoccupation with short-term returns, and the stultifying effect of the tunnel vision that comes with political agendas. Nowotny’s most important insights are that as a consequence of these commercial and political pressures, too much present-day research activity is goal oriented, in a narrow sense; and that excessive concentration on goals is pernicious, because it militates against serendipity, those unexpected discoveries that open up new possibilities that could not have been envisaged at the outset of the program that produces them.

The book comes with endorsements from several respected American academics, and for those whose professional interests or public responsibilities lie at the interface between science, politics, and economics, it could be said to be required reading. But for those who do not usually swim in these waters, a word of warning is in order. Nowotny is no ivory-tower academic: Every page of the book bespeaks the breadth of her reading, her interests, and her understanding of the political and commercial realities underlying the issues she explores. But she *is* a philosopher, and her long involvement in what one might call the sociology of science shows in the occasionally too-dense language in which her arguments are phrased. This makes her book rather hard going for the nonspecialist, and those with only an amateur interest in its subject matter may find the reading of it an unrewarding experience.

## Figures and Tables

**Figure:**